# The NHance^®^ Mutation-Equipped Anti-MET Antibody ARGX-111 Displays Increased Tissue Penetration and Anti-Tumor Activity in Advanced Cancer Patients

**DOI:** 10.3390/biomedicines9060665

**Published:** 2021-06-10

**Authors:** Philippe Aftimos, Christian Rolfo, Sylvie Rottey, Philippe Barthélémy, Christophe Borg, Keunchil Park, Do-Youn Oh, Sang-We Kim, Natalie De Jonge, Valérie Hanssens, Karen Zwanenpoel, Carla Molthoff, Daniëlle Vugts, Torsten Dreier, Peter Verheesen, Guus A.M.S. van Dongen, Julie Jacobs, Luc Van Rompaey, Anna Hultberg, Paolo Michieli, Patrick Pauwels, Samson Fung, Alain Thibault, Hans de Haard, Nicolas Leupin, Ahmad Awada

**Affiliations:** 1 Medical Oncology Clinic, Institut Jules Bordet, Université Libre de Bruxelles, 1000 Brussels, Belgium; philippe.aftimos@bordet.be (P.A.); ahmad.awada@bordet.be (A.A.); 2 University Hospital Antwerp, 2650 Edegem, Belgium; Christian.rolfo@umm.edu (C.R.); karen.zwaenepoel@uza.be (K.Z.); patrick.pauwels@uza.be (P.P.); 3 University Hospital Ghent, 9000 Ghent, Belgium; sylvie.rottey@ugent.be; 4 Medical Oncology Unit, Hôpitaux Universitaires de Strasbourg, 67000 Strasbourg, France; philippe.barthelemy@inserm.fr; 5 Medical Oncology Department, University Hospital of Besançon, CEDEX, 25000 Besançon, France; christophe.borg@efs.sante.fr; 6 Samsung Medical Center, Sungkyunkwan University School of Medicine, Seoul 06351, Korea; keunchil.park@samsung.com; 7 Seoul National University Hospital, Seoul 03080, Korea; ohdoyoun@snu.ac.kr; 8 Asan Medical Center, Department of Oncology, University of Ulsan College of Medicine, Seoul 05505, Korea; swkim@amc.seoul.kr; 9 Argenx BV, Industriepark Zwijnaarde 7, 9052 Ghent, Belgium; ndejonge@argenx.com (N.D.J.); vhanssens@argenx.com (V.H.); torsten.dreier@agomab.com (T.D.); pverheesen@argenx.com (P.V.); jjacobs@argenx.com (J.J.); lvanrompaey@dualyx.com (L.V.R.); ahultberg@argenx.com (A.H.); samson.fung@hotmail.de (S.F.); athibault23@gmail.com (A.T.); hdehaard@argenx.com (H.d.H.); 10 Department of Radiology & Nuclear Medicine, VU University Medical Center Amsterdam, 1012 Amsterdam, The Netherlands; cfm.molthoff@amsterdamumc.nl (C.M.); d.vugts@amsterdamumc.nl (D.V.); gams.vandongen@amsterdamumc.nl (G.A.M.S.v.D.); 11 AgomAb Therapeutics NV, 9000 Ghent, Belgium; paolo.michieli@agomab.com; 12 Department of Oncology, University of Torino Medical School, 10124 Turin, Italy

**Keywords:** MET, 18F-FDG-PET/CT, antibody, cancer

## Abstract

Dysregulation of MET signaling has been implicated in tumorigenesis and metastasis. ARGX-111 combines complete blockade of this pathway with enhanced tumor cell killing and was investigated in 24 patients with MET-positive advanced cancers in a phase 1b study at four dose levels (0.3–10 mg/kg). ARGX-111 was well tolerated up to 3 mg/kg (MTD). Anti-tumor activity was observed in nearly half of the patients (46%) with a mean duration of treatment of 12 weeks. NHance^®^ mutations in the Fc of ARGX-111 increased affinity for the neonatal Fc receptor (FcRn) at acidic pH, stimulating transcytosis across FcRn-expressing cells and radiolabeled ARGX-111 accumulated in lymphoid tissues, bone and liver, organs expressing FcRn at high levels in a biodistribution study using human FcRn transgenic mice. In line with this, we observed, in a patient with MET-amplified (>10 copies) gastric cancer, diminished metabolic activity in multiple metastatic lesions in lymphoid and bone tissues by 18F-FDG-PET/CT after two infusions with 0.3 mg/kg ARGX-111. When escalated to 1 mg/kg, a partial response was reached. Furthermore, decreased numbers of CTC (75%) possibly by the enhanced tumor cell killing witnessed the modes of action of the drug, warranting further clinical investigation of ARGX-111.

## 1. Introduction

Abnormalities in the HGF/MET axis have been reported in a wide variety of solid and hematological malignancies and have been associated with poor patient prognosis [1,2,3]. HGF/MET signaling has also been shown to drive resistance to molecular therapies directed against other oncogenic targets, including EGFR, B-RAF, HER2 and VEGFR [4]. Different mechanisms of MET activation have been described in human cancer including protein overexpression, gene amplification, point mutation, exon 14 deletion and paracrine or autocrine activation via HGF [1]. A variety of drugs have been designed to tackle these mechanisms [5]. The clinically most relevant drug types comprise small molecule agents targeting MET kinase activity [6] and antibodies interfering with HGF binding to MET and/or promoting receptor down-regulation [7]. Pre-clinical and clinical evidence suggests that HGF/MET inhibitors display cytostatic rather than cytotoxic activity on tumor cells [8,9].

ARGX-111 is the first anti-MET agent to be generated capable of killing MET-expressing tumor cells in addition to blocking HGF/MET signaling [10]. ARGX-111 is a glycoengineered germlined anti-MET mAb that combines four distinct mechanisms of action: (i) blockade of HGF-dependent signaling by ligand displacement; (ii) inhibition of HGF-independent MET signaling by receptor down-regulation; (iii) induction of cytotoxicity against MET-positive tumor cells via enhanced ADCC due to afucosylation in the Fc domain; and (iv) enhanced tumor targeting.

The anti-tumor effects of ARGX-111 have been extensively characterized in mouse models of cancer [10]. These experiments revealed that the cytotoxic function of ARGX-111 via its boosted ADCC potential is crucial for depleting circulating tumor cells (CTCs) and eliminating metastasis-initiating cancer stem cells, which an ADCC-impaired version of the antibody could not achieve.

ARGX-111 is equipped with NHance^®^ mutations in its Fc tail, which considerably increase the binding affinity to the neonatal Fc receptor (FcRn) in the acidic environment of the endosome, whereas the HN mutant binds with only very low affinity to human FcRn at neutral pH [11]. As a consequence, the Fc mutated antibody is rescued more efficiently from degradation than antibodies with wild-type Fc, leading to increased recycling or transcytosis. The better recycling translates into a prolonged serum half-life as was observed for, e.g., YTE and Xtend mutated antibodies [12], whereas improved transcytosis results in a more efficient transport into the tissues [13].

While most conventional MET-targeted antibodies blocking HGF/MET signaling displayed a safe profile in phase 1 clinical trials [14], the safety of an ADCC-enhanced anti-MET antibody with increased affinity to FcRn at acidic pH has never been tested in humans. Preclinical transcytosis assays and in vivo experiments in human FcRn transgenic mice to examine the transcytosis efficacy and targeting of FcRn-expressing organs are presented as well as the results of a first-in-human phase 1b study. The major aims were to determine the clinical safety of ARGX-111 in patients bearing tumors overexpressing the MET protein and/or harboring MET amplification, pharmacokinetics, pharmacodynamics and preliminary evidence of anti-tumor activity.

## 2. Materials and Methods

### 2.1. Transcytosis Assay with MDCKII Cells Expressing Human FcRn

Transwell filters (0.6 cm^2^) with polycarbonate-coated membranes and 0.4 µm pore size were incubated for 10 min in complete growth medium followed by seeding of 30,000 cells per well. Cells with passage number below 10 were used for all experiments. Transepithelial electrical resistance (TEER) was monitored using a Millicelll-ERS-2 V-ohm meter(Milipore, MA, USA). The cultures were grown for 14 days before reaching confluence with a TEER value of 375–425 Ωcm^2^. Prior to experiments, the monolayers were starved for 1 h in Hank’s Balanced Salt Solution (HBSS). Then 30 µg/mL MET antibodies, 300 µL diluted in HBSS pH7.4, were added to the apical chamber, and 600 µL HBSS pH7.4 was added to the basolateral compartment. Plates were incubated at 37 °C/5%CO_2_, and samples were collected from the basolateral compartment after 1 h, 2 h, 4 h and 6 h. Levels of transported IgG1 were quantified in the MET PK ELISA described below.

### 2.2. Quantification of IgG Transport by ELISA

Ninety-six-well MaxiSorp plates (NUNC) were coated with 2.5 µg decoy MET, diluted in PBS. Plates were blocked with 2% BSA fraction V for 2 h at room temperature, followed by washing 3 times with PBS/Tween20. Samples collected during transcytosis experiments were added undiluted and incubated for 2 h at room temperature before washing for 3 times. Captured MET antibodies were detected using HRP-conjugated goat anti-human IgG Fc. Binding was visualized by addition of 100 µL 1-step ABTS substrate. The reaction was stopped with 100 µL 1% SDS, and absorbance was recorded at 405 nm using a spectrophotometer (Tecan, Männedorf, Switzerland). The amount of transported IgG was determined from standard curves of each individual antibody.

### 2.3. Preclinical In Vivo FcRn Experiments

Mouse experiments were approved by the local ethics committee at VU University Medical Center, Amsterdam, the Netherlands.

ARGX-111 tissue distribution was explored using 32TG human FcRn transgenic mice (hFcRn Tg mice) and FcRn knockout mice with homozygous expression of human FcRn under an endogenous promotor [15]. The expression profile is similar to the human’s [16]. ARGX-111 is an afucosylated IgG1 with H433K/N434F substitutions in the CH3 domain to increase affinity to FcRn at pH 6.0 called NHance^®^ technology [11]. 53E2 is the germlined, fucosylated and non-mutated version of ARGX-111. For ^89^Zr labeling of ARGX-111 and 53E2, desferrioxamine (DFO/Df) was coupled to the protein followed by labeling with ^89^Zr. First, ARGX-111 and 53E2 were exchanged from their original buffer to 25 mM NaOAc + 50 mM NaCl pH 5.5 using 10kDA centrifugal filter units and centrifugation followed by modification with Fe-TFP-*N*-suc-Df as the chelator at a basic pH of ~9.5, followed by removal of iron at pH: 4.2–4.5 with EDTA. Labeling of ARGX-111- and 53E2-N-suc-Df with ^89^Zr was performed at pH: 6.8–7.2 with Hepes buffer for 60 min at room temperature in a volume of 2 mL. ^89^Zr-ARGX-111 and ^89^Zr-53E2 were successfully purified via PD-10 column with 25 mM NaOAc + 50 mM NaCl pH: 5.5 as the eluent. The fractions that contained the product were pooled and analyzed for their stability and radiochemical purity (HPLC, iTLC).

Three mice/group were injected retro-orbitally with 100 µg/3 MBq [^89^Zr]Zr-ARGX-111 or ^89^Zr-53E2 (without NHance^®^) with or without 4 mg human IgG (hIgG) preloading to establish a circulating IgG level in the normal range for humans. Mice were sacrificed 168 h and 336 h post infusion, and activity in the organs was counted using HPLC and iTLC and corrected to the total of radioactivity injected per mice and per organ weight.

### 2.4. Clinical Study Design

This was an open-label, multi-center, non-randomized, first-in-human Phase 1b study conducted in patients with MET-positive advanced malignancies. The primary objective of the study was to determine the recommended phase 2 dose (RP2D) of ARGX-111. Secondary objectives were to assess the safety, pharmacokinetics, immunogenicity, pharmacodynamics and preliminary antitumor activity of the antibody. The study consisted of two parts: a dose escalation (DE) phase followed by a safety expansion (SE) phase.

The DE phase consisted of four dose levels (0.3, 1, 3 and 10 mg/kg) administered Q3W. An accelerated titration scheme allowed one intra-patient DE per cohort [17]. The minimum number of patients treated at each dose level was two. The dose of ARGX-111 would be escalated until the occurrence of a dose-limiting toxicity (DLT) observed in any of the first patients recruited to a given cohort. At that point, a total of 6 patients would be recruited to the dose level at which a DLT was observed. If no additional DLT was observed at that dose level (i.e., DLT in 1/6 patients), DE could proceed. If two or more DLTs were observed at any dose level (i.e., DLT in ≥2/6 patients), DE would stop, and that dose would be determined to have exceeded the MTD. Three additional patients would then be recruited to the immediately lower dose level. If the incidence of DLT did not exceed 1 out of 6 patients, that dose level would define the MTD.

A [^18^F]FDG-PET/CT scan was performed at screening (max. 7 days before first dose) and at cycle 2 day 15 (C2D15). Patients whose PET/CT scan demonstrated decreased metabolic activity would continue treatment at the same dose according to RECIST 1.1. Patients whose PET/CT scanning demonstrated stable or increased metabolic activity would be treated at the next higher dose from C3D1 and beyond, unless precluded by toxicity (drug-related, Grade 3 or 4 severity). As a result, a single intra-patient DE was allowed for patients initially treated at the 0.3, 1 and 3 mg/kg dose levels. Patients who underwent intra-patient DE had a repeat PET/CT at C4D15 (Figure S4).

The SE phase would be conducted using a dose of ARGX-111 equal to or lower than the MTD determined during the DE of the study. The dose selection would be based primarily on the safety and PK profiles of ARGX-111. No intra-patient dose escalation was allowed in the SE phase of the study. The patients would stay on the study until they developed progressive disease (PD) or intolerable drug-related toxicity or withdrew consent to receive further treatment, for any reason.

The trial (EudraCT 2013-002901-72) was conducted in compliance with the Declaration of Helsinki and the ICH E6 GCP Guidelines. Each patient provided written informed consent before pre-screening and enrollment. The clinical study protocol and its amendments, informed consent documents and any other appropriate study-related documents were reviewed and approved by the applicable regional review boards or ethical committees.

### 2.5. Patients and Eligibility Criteria

For the DE phase, prescreening was performed by IHC (validated using an anti-MET β-chain antibody; Immuno-Biological Laboratories, Gunma, Japan) on a pretreatment biopsy or archived tumor sample. Patients demonstrating MET overexpression (>50% tumor cells positive, intensity ≥2+) were subjected to a PET/CT scan. Condition for further study consideration was at least one tumor lesion larger than 2 cm with an FDG uptake at least 1.5 times greater than the mean liver standardized uptake value (SUV) plus 2 standard deviations. For the SE phase, prescreening was performed by FISH (MET probe, Kreatech MET probe (Leica Biosystems, Diegem, Belgium)) on a pre-treatment biopsy or archived tumor sample. Patients with solid tumors demonstrating *MET* amplification, defined as a *MET*/*CEP7* ratio ≥2, were further screened for eligibility.

For both phases of the study, all eligible patients had to meet the following criteria: be ≥ 18 years old; have an Eastern Cooperative Oncology Group (ECOG) performance status of 0 or 1; be relapsing and/or refractory to prior cancer therapy. Eligibility criteria also included: serum albumin > 35 g/L; absolute neutrophil count > 1.0 × 10^9^/L; hemoglobin > 90 g/L; platelet count ≥ 75 × 10^9^/L; activated partial thromboplastin time ≤1.5 × ULN; total bilirubin ≤ 1.5 × ULN; creatine phosphokinase ≤ 2.5 × ULN; serum creatinine ≤ 1.5 × ULN.

Patients were excluded if they had a history or clinical evidence of CNS involvement (however, irradiated brain metastases that had been stable for >1 month and did not require systemic glucocorticoid administration were allowed), major surgery or biological therapy (monoclonal antibodies) 4 weeks prior to ARGX-111 first dose administration. Patients were also excluded if they had 3 weeks prior to ARGX-111 first dose administration: systemic glucocorticoids at doses greater than physiological replacement (prednisone 20 mg equivalent); cytotoxic chemotherapy; or radiation therapy with curative intent. Further criteria for exclusion included: biological therapy other than monoclonal antibodies within 5 half-lives of ARGX-111 first dose administration; unresolved Grade 3 or 4 toxicity from prior therapy, including experimental therapy; history of recurrent Grade 3 or 4 toxicity from anti-MET therapy; uncontrolled diabetes, defined as fasting glycemia >150 mg/dL; active, untreated viral, bacterial or systemic fungal infection; any clinical finding, including psychiatric and behavioral problems; childbearing potential (unless an adequate measure of contraception was used); pregnancy or lactation; history of severe (Grade 3 or 4) hypersensitivity to recombinant proteins.

### 2.6. Rationale for Dose Selection and Treatment

The highest ARGX-111 dose used in the DE was calculated based on a NOAEL of 30 mg/kg observed in cynomolgus monkeys (*Macaca fascicularis*), which corresponds to a human equivalent dose (HED) of 10 mg/kg. The lowest dose used in the DE was determined based on the minimal effective dose observed in mice (1.5 mg/kg) and PK data obtained in monkeys, which indicated that a dose of 0.3 mg/kg was expected to be associated with human serum concentrations that are in the range of pre-clinical activity for less than 1 week. The same PK data also suggested that the highest dose maintained effective drug concentrations for at least 3 weeks. Based on these observations, a dosing interval of Q3W was chosen for the DE phase. For the SE phase, a dose of 3 mg/kg (MTD) was selected based on the results obtained in the DE phase. A dosing interval of Q2W was chosen because Q3W would have resulted in suboptimal drug concentration during the third week. To minimize overdosing risk, the dose of ARGX-111 in the SE phase was capped in patients weighing >80 kg.

Patients who met the eligibility criteria of the study were hospitalized for a minimum of 24 h to receive their first dose (C1D1) of ARGX-111. A pre-medication regimen was designed to reduce the incidence of infusion-related reactions (IRRs), often associated with the administration of ADCC-enhanced antibodies [18]. Approximately 12 h prior to each ARGX-111 administration, patients were given the following therapy, depending on institutional practice: anti-pyretic (e.g., acetaminophen 1000 mg); H1 blocker (e.g., diphenhydramine 50 mg or promethazine 25 mg); H2 blocker (e.g., ranitidine 50 mg or famotidine 20 mg); glucocorticoid (e.g., methylprednisolone 32 mg). Approximately 30 min prior to antibody infusion, the same therapy was repeated except that glucocorticoid dosing was increased (e.g., methylprednisolone 60 mg). ARGX-111 was administered via an IV infusion in stepwise increments.

### 2.7. Safety and Tolerability

Safety and tolerability were assessed through clinical and laboratory evaluations at weekly interval for the first cycle and, thereafter, Q3W for the DE and Q2W for the SE. Adverse events (AEs) were monitored since the patient signed the screening informed consent and continued up to date of end-of-study (EOS) visit. AEs ongoing at EOS were continued to be monitored until resolution or 60 days post EOS. AEs were graded according to NCI-CTCAE Version 4.03. Dose-limiting toxicity (DLT) was defined as a drug-related, Grade 3 or 4 AE occurring during the 21 days following the first dose of ARGX-111.

### 2.8. Response Assessment

Tumor response was assessed using appropriate methods including CT scan, MRI, chest X-ray and/or PET/CT, depending on tumor type. All patients who had completed one cycle of therapy and undergone one scheduled tumor assessment were considered evaluable for response by the investigator. Tumor response was recorded as the best response achieved and was expressed as % change compared to baseline measurement. Patients were evaluated for response according to RECIST 1.1.

### 2.9. Pharmacokinetics and Additional Assessments

In the DE phase, serum samples were collected at the following time points: for all cycles, up to 8 h prior to infusion start (pre-samples); on C1D1, at 0, 2, 6 and 12 h post-infusion; on C1D2, D8 and D15; for C2 and beyond, at 0 h post-infusion. Time “0” refers to end of infusion. In the SE phase, serum samples were collected up to 8 h prior to infusion start (pre-samples) and the end of infusion (post-samples) for all cycles. ARGX-111 serum concentration was determined using a validated ELISA method employing soluble extracellular MET domain in solid phase (outsourced to IPM Biotech, Hamburg, Germany). The lower limit of quantification (LLOQ) of the assay was 0.2 µg/mL; the upper limit of quantification (ULOQ) was 15 µg/mL.

Anti-drug antibodies (ADAs) were determined by enhanced chemiluminescence (ECL) using a homogeneous bridging format assay (IPM Biotech). The LLOQ of the assay was 8.3 ng/mL; the ULOQ was 13,800 ng/mL. For CTC measurement, whole blood samples were collected at screening for the DE phase. CTCs were measured using the CellSearch technology (Veridex LLC, Raritan, NJ, USA). CTC number was followed up during treatment in patients showing significant CTC number (>3 CTCs/7.5 mL). Serum HGF was measured in pre-samples for all cycles of the DE phase and in both pre- and post-samples for all cycles of the SE phase using an ELISA kit (R&D Systems, Minneapolis, MN, USA). NK cell count and function were analyzed on whole blood samples collected on pre-samples for all cycles of the DE and SE phases and on C1D2 of the DE phase. NK cells (CD45^+^/CD3^−^/CD16^+^/CD56^+^) were counted using Trucount tubes (BD Biosciences, San Diego, CA, USA) and a FACSCanto apparatus (BD Biosciences). NK function was analyzed on NK cells isolated using RosetteSep™ Human Enrichment Cocktail in combination with RosetteSep™ DM-L Density Medium (STEMCELL Technologies, Vancouver, Canada) and counted using BD TriTest™ CD3-FITC/CD16^+^CD56-PE/CD45-PerCP (BD Bioscience) to determine absolute numbers. L540 cells (Life Technologies, Carlsbad, CA, USA) were mixed with NK cells (5000:50,000); 50 µg/mL ARGX-111 or IgG1 isotype control was added and incubated for 24 h at 37 °C in 5% CO_2_. After washing, anti-human CD30 PE (eBioscience, San Diego, CA, USA) was added, and the lysis of L540 cells was measured using a FACSCanto (BD Biosciences) and calculated as percentage lysis as compared to L540 cells alone.

### 2.10. Statistical Methods

All statistical analyses were performed using SAS software 9.3 or higher (SAS Institute, Cary, NC, USA) and GraphPad Prism (version 7 and 8, Graphpad, San Diego, CA, USA). Demographic, baseline characteristics, PK, PD and preliminary tumor assessment were summarized using mean, standard deviation and/or median and range. Safety data were summarized for the complete population (DE + SE).

## 3. Results

### 3.1. Characterization of ARGX-111

**Figure 1:**
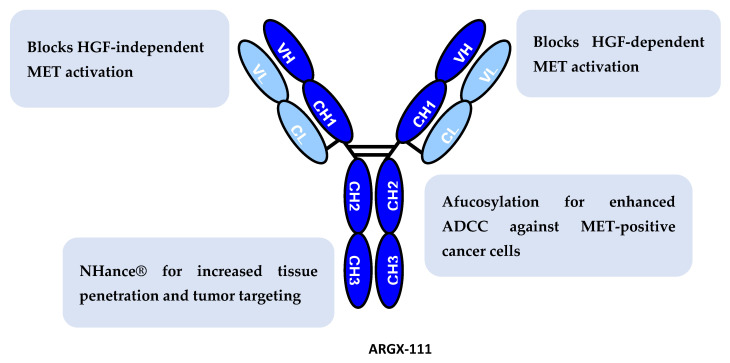
Modes of Action of ARGX-111. ARGX-111 combines 4 distinct mechanisms of action: (1) it potently competes with HGF for MET binding, thus inhibiting ligand-dependent MET activity; (2) it induces receptor down-regulation, thus curbing HGF-independent MET activity; (3) it engages NK cells to kill MET-expressing cancer cells, thus displaying MET-specific cytotoxic activity; and (4) it has increased tumor targeting and penetration through interaction with FcRn.

ARGX-111 is a germlined llama-derived antibody, which potently blocks both HGF-dependent and -independent signaling (Figure 1). Considerable efforts had to be undertaken to identify this lead from a large panel of antibodies that in its bivalent IgG format was able to antagonize the HGF-independent pathway without inducing cross-linking, i.e., agonizing, the MET receptor [19].

The antibody is glycoengineered by the POTELLIGENT^®^ technology and is produced in the CHOK1SV cell line, which lacks α1,6-fucosyltransferase 8, the enzyme that adds the terminal fucose to the N-linked carbohydrate moiety of the Fc. The afucosylated antibody has a higher affinity for FcγRIIIa on natural killer cells leading to more potent ADCC and with unchanged affinity to FcγRI, while the ADCP via macrophages was not affected [10]. ADCP due to increased affinity for FcγRIIIb on neutrophils could potentially be increased but needs to be further investigated [20].

**Figure 2:**
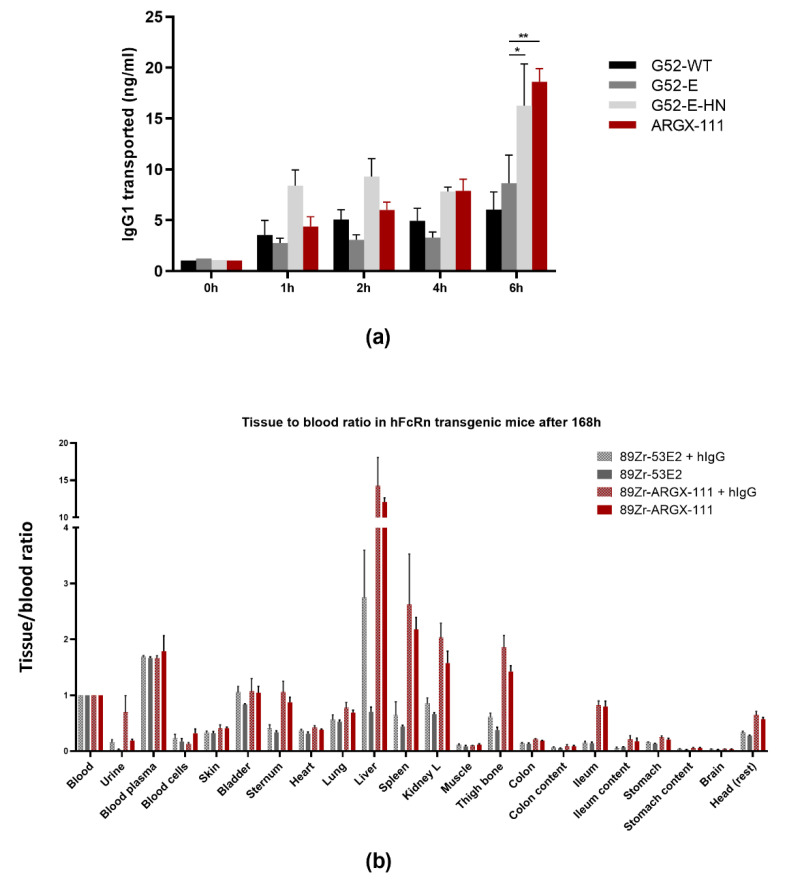
In vitro and in vivo characterization of NHance^®^-mutated ARGX-111 for FcRn-mediated transcytosis. (**a**) NHance mutations promote transcytosis across MDCK II cells overexpressing human FcRn. Transwell assay showing apical-to-basolateral transport of Fc-engineered variants of ARGX-111. Measured antibody concentrations in the basolateral compartment are shown in ng/mL; (**b**) Tissue distribution of ARGX-111 in 32TG human FcRn transgenic mice. Tissue/blood ratio in mice (N = 3) analyzed 168 h after administration of ^89^Zr-labelled ARGX-111 or 53E2 in the presence of hIgG or not. Bars represent mean ±SEM; * *p* = 0.0174; ** *p* = 0.0005.

The antibody harbors the NHance^®^ mutations H433K/N434F in the CH3 domain of the heavy chain yielding an increased affinity for binding to FcRn at pH 6.0 [10]. To evaluate the potency in transcytosis, a similar type of assay was performed as described previously for NHance^®^-equipped antibodies [11,21]. Madine–Darby canine kidney II (MDCKII) cells stably transfected with human FcRn were grown on Transwell filters, and ARGX-111 and its variants G52-E-NH, G52-E and G52 were investigated. The molecules G52-E-NH and G52-E are identical to ARGX-111, but instead of the afucosylated Fc, they contain the mutations S239D/I332E that in contrast to afucosylation increase the affinity to both human FcγRIIIa and its mouse orthologue FcγRIV, thereby better mimicking the ADCC-induced cell killing potential in murine disease models. The ARGX-111 variants were added to the apical reservoir, and at various time points, samples at the basolateral side were taken for measurement of transport across the MDCKII cells overexpressing human FcRn. Three independent experiments were performed and quintuplicate samples were taken for each antibody at different time points of 1, 2, 4 and 6 h. Each of the antibodies was transported in a time-dependent manner, and NHance^®^-equipped antibodies ARGX-111 and G52E-NH were transported more efficiently than G52-E and G52-WT (Figure 2a). Between experiments, ARGX-111 was transported 1.8 ± 0.5 and 2.4 ± 0.2-fold (average ± SD) more efficiently than G52-E after 4 and 6 h, respectively, and G52-E-NH was transported 1.7 ± 0.6 and 2.2 ± 0.3-fold (average ± SD) more efficiently than G52-E after 4 h and 6 h, respectively. Thus, the NHance^®^ equipped antibodies showed more efficient FcRn-dependent transepithelial transport.

To explore the tissue targeting potential as a consequence of the FcRn-induced transport into the tissues, a biodistribution study in the hemizygous human FcRn transgenic mouse strain Tg32 [15] was performed. To this end, ARGX-111 and G52-WT were modified with the chelator Fe-TFP-*N*-suc-Df leading to incorporation of 1.24 chelator molecules per antibody molecule. Subsequently labeling with ^89^Zirconium (^89^Zr) was performed resulting in an antibody preparation with a specific activity of 30.0 MBq/mg for ARGX-111 and G52-WT. Human FcRn transgenic mice were injected with ^89^Zr ARGX-111 and G52-WT (3 MBq, 100 μg) with or without prior administration of human IgG (4 mg) to compensate for the lower amounts of circulating mouse IgG, which these transgenic animals have because of the low affinity of mouse IgG for human FcRn. The NHance^®^-equipped ARGX-111 showed increased uptake in liver but also in the lymphoid organs spleen, ileum, thigh bone and sternum, while G52 was not detected in any of these tissues (Figure 2b). ARGX-111 does not cross-react with mouse MET, and thus the uptake was assumed specific for NHance^®^ on ARGX-111.

### 3.2. Patient Demographics and Baseline Characteristics

Between 24 February 2014 and 13 September 2016, a total of 24 patients were enrolled and treated in the study, 19 in the Dose Escalation (DE) and 5 in the safety expansion (SE) phase. A total of 192 patients were prescreened for MET expression by IHC, of which 70 (36%) matched the positivity criteria for the DE (>50% cells expressing MET *AND* 2+/3+ intensity). Gastric, esophagus and kidney cancers displayed the highest positivity (63%) for MET expression (Figure S1A). Of these 70 positive patients, 19 matched all the eligibility criteria and were included in the DE phase.

In the SE, a total of 428 patients were prescreened for *MET* amplification by FISH, of which 15 (3.5%) matched the positivity criteria for the SE (*MET/CEP7* ratio ≥ 2). Lung and gastric cancers displayed the highest frequency of *MET* amplifications (Figure S1B). Of these 15 positive patients, 5 were eligible and were enrolled in the SE phase. The archived biopsies of the SE patients were further analyzed by IHC. This analysis revealed that the tumors bearing high *MET* copy number (≥5) also displayed a high percentage of cells expressing MET (≥80%) and high MET expression levels (intensity ≥ 2; Table S1).

No clinically relevant difference regarding demographic characteristics was observed when comparing the treatment groups (Table 1).

### 3.3. Dose Escalation and Dose-Limiting Toxicity

A total of 19 patients in the DE phase were enrolled and treated Q3W: 0.3 mg/kg cohort, N = 2; 1 mg/kg, N = 2; 3 mg/kg, N = 12; and 10 mg/kg, N = 3. Intra-patient dose escalation at C3 was allowed based on PET/CT on C2D15 as per protocol. In the 0.3 mg/kg cohort, one gastric cancer patient was escalated to 1 mg/kg. In the 1 mg/kg cohort, one esophagus cancer patient was escalated to 3 mg/kg. In the 3 mg/kg cohort, one cervix cancer patient was escalated to 10 mg/kg. Two DLTs were observed in the 10 mg/kg cohort. One kidney cancer patient and one cervix cancer patient experienced a Grade 3 IRR and were de-escalated to 3 mg/kg from C2D1. As per protocol, an additional group of three patients was recruited to the immediately lower dose cohort (3 mg/kg). No DLT was observed in these patients. As an extra precaution, seven additional patients were enrolled in the same cohort, without showing any DLT. The MTD was therefore established at 3 mg/kg. Based on these results and on PK data from the DE phase (see below), the dose of 3 mg/kg (MTD) administered Q2W was chosen for the SE phase.

### 3.4. Safety and Tolerability

ARGX-111 was generally well tolerated up to 3 mg/kg and had an overall favorable safety profile. An overview of the treatment-emergent adverse events (TEAEs) in both DE and SE are shown in Table 2. A total of 263 TEAEs were experienced by all 24 patients during the study (DE + SE). Of these, the majority were CTCAE Grade 1 (141/263). Overall, 125 TEAEs (48%) were considered related to ARGX-111. The most commonly reported adverse events were IRRs, affecting 80% of the patients, the majority of them classified as CTCAE Grade 2. Pre-medication was administered as per institutional practice prior to each treatment cycle to manage the clinical manifestations of IRR. In total, five TEAEs of CTCAE Grade 3 intensity were considered related to ARGX-111, all of which occurred during DE: one patient in the 3 mg/kg Q3W cohort with three events (back pain, pain in extremity and fatigue) and two patients in the 10 mg/kg Q3W cohort with one event each (both IRRs). There was no trend of specific symptoms for IRRs between patients, and they were all resolved. No significant difference in TEAEs was observed for patients who were dose-escalated. The relatively mild nature of most TEAEs and the low frequency of drug-related CTCAE Grade 3 events suggest an acceptable safety profile for the ARGX-111 cohorts investigated during the study.

Overall, 27 serious adverse events (SAEs) were reported, including 19 events in the DE and 8 in the SE. Of these, 8 SAEs were considered related to ARGX-111 (7 in the DE and 1 in the SE), including 7 IRRs and 1 bone pain. All related SAEs were resolved during the course of the study. A total of 8 patients died during the study (7 in the DE and 1 in the SE). Disease progression and underlying disease were the cause of death for all patients who died, as can be expected in this patient population with advanced cancer.

Generally, there was no significant trend or notable influence of ARGX-111 on the laboratory results or on the vital signs over time during the study, with no notable difference between dose cohorts. There was no clear trend in any new abnormal physical examination findings developing during the study.

### 3.5. Pharmacokinetics and Anti-Drug Antibodies

**Figure 3:**
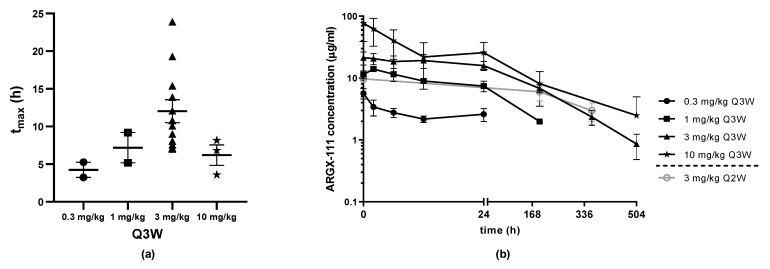
PK analysis of ARGX-111 at C1. (**a**) Mean serum concentration over time after IV infusion of ARGX-111 by dose level; (**b**) Mean time taken to reach peak serum concentration (t_max_) by dose level for DE (Q3W: 0.3 mg/kg (N = 2); 1 mg/kg (N = 2); 3 mg/kg (N = 12); 10 mg/kg (N = 3); Q2W: 3 mg/kg (N = 5)). Graphs represent mean ± SEM.

The PK parameters based on non-compartmental analysis indicated dose proportionality at the higher doses of 3 and 10 mg/kg and are summarized in Table 3. The median C_max_ in C1 was 5.65 µg/mL, 14.2 µg/mL, 25.2 µg/mL and 101 µg/mL for the 0.3 mg/kg, 1 mg/kg, 3 mg/kg and 10 mg/kg cohorts in the DE, respectively. In the SE (3 mg/kg), the C_max_ was not measured because the sample time points were 192 h and 360 h and the mean time taken to reach peak serum concentration (t_max_) for the different cohorts was around 12 h (Figure 3a). The median serum concentrations at 192 h were 9.10 µg/mL for the 3 mg/kg Q2W SE cohort and 6.38 µg/mL for the 3 mg/kg Q3W DE cohort. The mean ARGX-111 serum concentrations for the DE cohorts are shown in Figure 3b. The estimated half-life (t_1/2_) was around 5 days, and the volume of distribution was 11.5 L for the cohort of 12 patients dosed with 3 mg/kg Q3W and 14.3 L for the last group of 3 patients administered with 10 mg/kg Q3W. Intra-patient variability may be explained by low patient number in some cohorts and by variations in sampling times (due to infusion interruptions). There were no relevant anti-drug antibodies detected at any dose level in the DE (Figure S2). Limited immunogenicity analysis was performed in SE from which no conclusions can be drawn.

### 3.6. Exploratory Assessments of ADCC-Mediated Depletion of Circulation Tumor Cells (CTCs)

The number of CD16^+^/CD56^+^ NK cells was stable over the cycles analyzed for all patients. There was no significant (ANOVA test) decrease in ARGX-111-mediated lysis of L540 cancer cells using autologous NK cells over the cycles in any of the dose cohorts indicating that the number of NK cells and the activity remained during the treatment (Table S2). CTC samples were analyzed at screening in 10/19 patients enrolled in the DE, of which 7 were positive as determined by CELLSEARCH technology analysis. Overall, 3/7 positive patients displayed >3 CTCs/7.5 mL of blood. Two of these patients were off the study before C3 and were therefore not followed up for CTCs; the third patient (a metastatic gastric cancer patient who got dose-escalated from 0.3 mg/kg to 1 mg/kg) had the highest number of CTCs (112 CTCs/7.5 mL of blood) and started responding at C5. A CTC sample collected at C6 displayed a 75% reduction in CTC number compared to baseline.

### 3.7. Duration of Study, Preliminary Anti-Tumor Activity and Best Response

**Figure 4:**
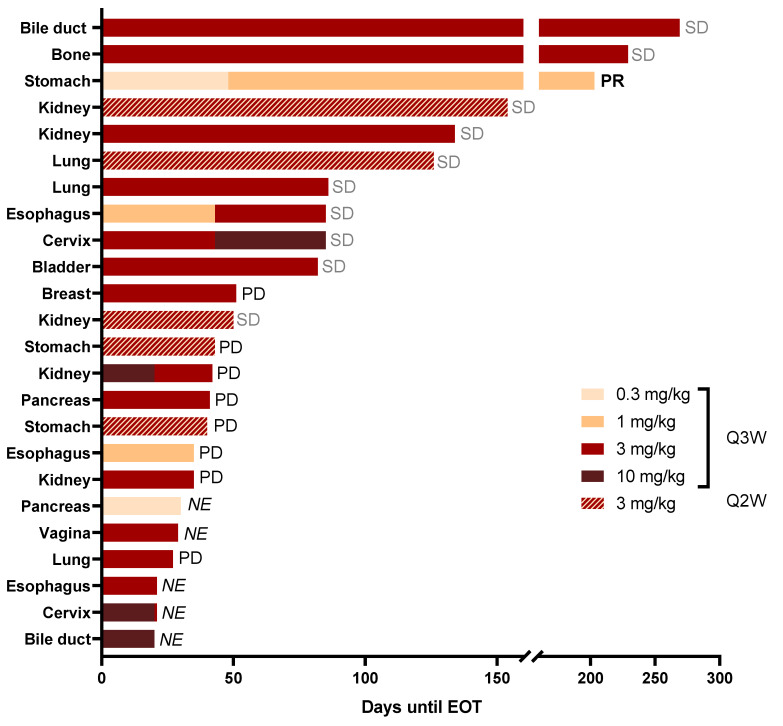
Mean duration of study and best response. Each bar represents one patient and the length of the bar the duration of treatment from until C1D1 end of treatment (EOT). The doses and the administration interval are indicated by the color code. The best response as per investigator review is also indicated on the right for each patient. PR, partial response; SD, stable disease; PD, progressive disease; NE, not evaluable according to RECIST 1.1.

All 24 patients completed the first cycle of ARGX-111 treatment. The mean duration of study was 81 days in the DE (11.6 weeks) and 84 days in the SE (12.0 weeks). The mean number of cycles was 3.9 for the DE and 6.0 for the SE. The longest duration was observed for a 68-year old female patient with bile duct cancer who had received one line of chemotherapy followed by laparotomy. The patient had one liver lesion when treated with 3 mg/kg ARGX-111 Q3W and stayed on study for 38 weeks, with stable disease (SD) as best response (Figure 4).

**Figure 5:**
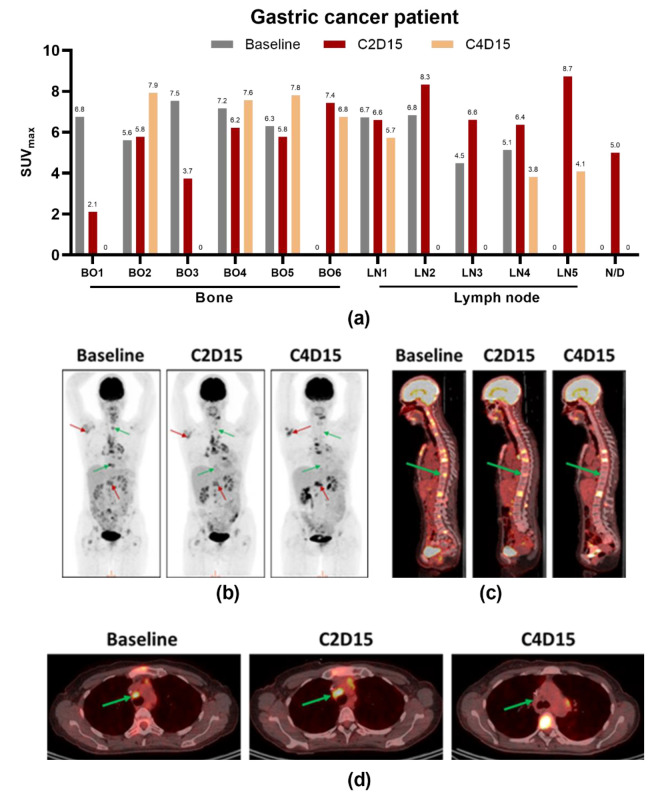
PET/CT scans of a *MET*-amplified gastric adenocarcinoma patient showing response to ARGX-111. (**a**) SUV_max_ values at baseline, C2D15 and C4D15 grouped by lesion (BO, bone; LN, lymph node; N/D, not determined); (**b**) Representative images of ^18^F-FDG uptake; (**c**) CT images in parasagittal view; (**d**) Axial view of a responding lymph node lesion at baseline, C2D15 and C4D15. Red arrows, non-responding lesions; green arrows, responding lesions.

Preliminary anti-tumor activity as per RECIST was observed at dose levels 0.3–3 mg/kg. The disease control rate (DCR) was 46% in the DE and SE, with 1/24 partial response (PR) and 10/24 stable diseases (SDs). In the SE phase, 3/5 patients had SD. Five patients in the DE were not evaluable for response (Figure 4). The best overall response as per investigator review was partial response (PR) in a 49-year old female with gastric cancer who had three prior lines of chemotherapy and total gastrectomy. She had nine measurable lesions in lymph nodes and bone at baseline when she started treatment with 0.3 mg/kg ARGX-111. Before C3, she showed progressive disease with signs of diminished metabolic response in some lesions by PET/CT and was therefore escalated to 1 mg/kg as allowed by protocol. At C4D15, she had another PET/CT which showed that four lesions completely disappeared (two in bone and the other two in lymph nodes). All the lymph node lesions responded to treatment. Three new lesions developed at C2, of which one disappeared at C4 (Figure 5a). Representative images of the PET/CT scans for this patient are shown in Figure 5b–d. Partial response was achieved at C6 according to RECIST that persisted until C9, meaning the patient was on the study for a total of 29 weeks before progressing. The PR was in line with the number of CTC reduced by 75% as compared to baseline. Retrospective analysis of her archived primary tumor tissue showed that she was highly *MET*-amplified (>10 copies).

The accelerated titration design of the study allowed one intra-patient dose escalation at C3 dependent on PET/CT imaging results at C2D15. These results were also used to explore early metabolic responses to treatment. Apart from the gastric cancer patient, one esophagus cancer patient was escalated from 1 mg/kg to 3 mg/kg, and one cervix cancer patient from 3 mg/kg to 10 mg/kg. These patients received three PET/CT scans: one at screening (baseline), one at C2D15 and one at C4D15. All escalated patients improved or stabilized, one PR and two SD after escalation to a higher dose level.

A 57-year female suffering from renal cell cancer with moderately *MET*-amplified tumors (7.5 copies, *MET/CEP7* ratio of 2.7) was treated in the SE cohort at 3 mg/kg Q2W. One lesion in the lymph node showed lower metabolic activity at cycle 9, which already was visible at cycle 3 (Figure S3). A lesion in the lung initially responded at cycle 3 but progressed at cycle 6.

## 4. Discussion

ARGX-111 is the only MET-targeting antibody with effector functions, which, in addition to antagonizing the receptor, actively kills cells by enhanced ADCC and ADCP based on the application of the POTELLIGENT^®^ technology [10,22,23]. ARGX-111 is also the only antibody targeting MET equipped with the NHance^®^ mutations that was shown to promote FcRn-mediated transcytosis, making tissues better accessible to the antibody.

The results presented in this study indicate that ARGX-111 monotherapy has an overall favorable safety and tolerability profile up to 3 mg/kg in heavily pre-treated patients with advanced MET-positive malignancies. Despite broad expression of MET on normal tissues, no drug-related severe toxicity was observed up to 3 mg/kg. NK cell count and function were preserved in all cohorts, suggesting that ADCC enhancement does not cause NK cell exhaustion or impairment. As commonly experienced with therapeutic antibodies and in particular with glycoengineered antibodies, IRRs represented a common drug-related AE [24,25]. With the exception of the two Grade 3 IRRs resulting in DLT at 10 mg/kg, all other IRRs were mild to moderate and not dose-dependent. In any case, all IRRs were manageable, limited in time and completely resolved by standard medication regimen and reduced infusion rates. Different route of administration such as subcutaneous could be considered to reduce the numbers of IRR as was shown for alemtuzumab and trastuzumab [26,27], but a clinical study with equivalent groups needs to be performed.

Based on the two DLTs observed at 10 mg/kg, the MTD was established at the next lower dose (3 mg/kg). Since PK data indicated that Q3W administration would have resulted in drug concentrations below those associated with the minimal effective dose in mice (1.5 mg/kg) for the third week of the cycle, a Q2W administration scheme was chosen for the SE phase. The safety, duration of study and preliminary anti-tumor activity data obtained indicate that this administration scheme is appropriate. However, this dosage could still result in sub-optimal ARGX-111 concentrations. Doses above 3 mg/kg could further be explored to determine the RP2D.

Preliminary anti-tumor assessment revealed a high DCR (46%) spread over a wide variety of metastatic cancers. This could be due to the four modes of action of ARGX-111, thus providing increased versatility against different mechanisms of MET dysregulation. Indeed, ARGX-111 could stabilize tumor growth in patients with multiple metastatic lesions, which are presumably heterogeneous from a genetic viewpoint. Although the best responder was a gastric cancer patient with a *MET*-amplified primary tumor, long-term stabilization was achieved in a bile duct cancer patient displaying a normal *MET* copy number but a high percentage of MET-expressing tumor cells and an expression intensity of 2+.

Several MET antagonists demonstrated efficacy in *MET*-amplified patients, but the challenge to identify these patients with the most appropriate method and criteria still remains uncertain. As with *HER2*-amplified gastric cancers, where a cutoff of *HER2*:*CEP17* ratio of >5 was defined for effective trastuzumab treatment [28], high-level *MET* amplification (*MET/CEP7* >5) was found to give a positive clinical outcome based on the results from a study with crizotinib in non-small cell lung cancer [18]. In light of the partial response seen of ARGX-111 in the gastric cancer patient it is of interest to notice that in this indication, responses have been observed in *MET*-amplified patients with several antagonists, such as the multikinase inhibitor crizotinib [29], the MET-specific kinase inhibitors AMG 337 [30] and SAR125844 [31] and the MET-targeting antibody ABT-700 [32]. In the ABT-700 study, 3/4 patients with *MET* amplification reached PR and had a longer PFS than any prior therapy. *MET* amplification was only detected in the metastatic recurrent tumors and not in the primary indicating that a treatment-refractory patient population should be targeted. *MET* amplification has been shown to be around 50% higher in the metastasis than the primary tumor in Chinese metastatic gastric patients [33] and NSCLC patients [34].

In 5 to 20% of patients’ resistance to EGFR treatment was associated with *MET* amplification, as was reported for therapy in CRC with antibodies [35] and in NSCLC for the kinase inhibitors erlotinib and gefitinib [36]. Increased MET expression as the result of gene amplification might lead to heterodimerization with and transphosphorylation of other tyrosine kinase receptors, such as EGFR, Her2, HER3 and RET explaining the escape via MET during therapies directed against these receptors [37]. More recently heterodimerization of MET with Poly (ADP-Ribose) Polymerase (PARP) was demonstrated preclinically to result in MET-mediated phosphorylation of this target and hence increased enzymatic activity of PARP. As a consequence, the efficacy of a PARP inhibitor was lost, which could be restored by combination treatment with a MET inhibitor [38]. Combination treatment of ARGX-111 with drugs targeting receptors such as mentioned above might prevent resistance.

As mentioned above, one of the challenges is to identify the low numbers of patients with *MET* amplification that potentially could benefit from MET-targeted therapy. FISH has been used in many studies, including our SE phase, but the major limitation is that one most often uses archived material from the primary tumor tissue for screening which may not reflect the status of the patients when they enter the study, i.e., refractory after prior therapy/therapies.

Detection of *MET* amplification using cell-free DNA and amplification by droplet digitally based PCR methods using circulating tumor DNA (ctDNA) isolated from plasma samples enabling cheap, fast and high throughput screening are being investigated [39,40,41,42]. Additionally, one could utilize a CTC technology to detect, capture and characterize MET-expressing CTCs. This would allow one to analyze MET overexpression, *MET* amplification or mutation and other mechanisms of MET activation in patients in real time as well as the number of CTCs [43].

Stabilization of disease in 10 out of 24 patients treated with ARGX-111 is remarkable, and 5 of these patients were in the study for more than 126 days. Eighteen of these patients did not have *MET*-amplified tumors but had strong overexpression as was demonstrated by IHC. The rationale of enhanced cell killing was also applied with the Antibody Drug Conjugate ABBV-399 directed against MET, which in pre-clinical experiments gave efficacy in MET-overexpressing but not gene-amplified tumor cells in contrast to the parental antibody ABT-700 that only had therapeutic activity in *MET*-amplified tumors [44]. Our previous data generated in pre-clinical models demonstrated that the ADCC function of ARGX-111 is crucial for depleting CTCs and metastasis-initiating cancer stem cells [10]. Consistent with this, a very significant (75%) CTC reduction was observed in the best-responding gastric cancer patient. Unfortunately, no other patient was informative with respect to CTCs because of either a too-low baseline number or early EOT. The number of CTCs could represent a useful marker for monitoring therapeutic response to ARGX-111. The results obtained with PET/CT imaging provide evidence that measuring tumor metabolic activity may be an effective approach to assess early response to MET inhibition [45]. While HGF/MET signaling has been traditionally implicated in tumor cell proliferation, survival, motility and epithelial-to-mesenchymal transition, recent data point at its pivotal role in controlling glucose metabolism in cancer cells [46,47]. The effects of MET blockade on glucose uptake are likely to be more rapid and immediate compared to its anti-proliferative effects, which may take weeks to occur.

In the biodistribution studies of human FcRn transgenic mice, accumulation of radiolabeled ARGX-111 was found in lymph nodes and bone, tissues known to have high FcRn expression [48]. In the gastric cancer patient, a decreased metabolic activity in the metastatic lesions in lymph nodes and bone could be observed by PET/CT imaging after treatment with a dose as low as 0.3 mg/kg. Also in the renal cell cancer patient, this type of response occurred in a lesion in one of the lymph nodes. The NHance^®^ mutations promote transcytosis via FcRn and thereby improve transport of ARGX-111 from the periphery into tissues expressing elevated levels of this receptor. The targeting of high-expressing FcRn organs including liver is of interest for therapeutic applications. Since MET is also found on hepatocytes, we monitored for side effects associated with liver but could not find any sign of toxicity in the treated patients. Especially for liver cancer or metastatic lesions in this organ but also viral diseases like hepatitis, the NHance^®^-mediated targeting has the potential to improve the therapeutic efficacy. Accumulation of an Fc engineered anti-VEGF antibody in the acidified environment of tumors expressing FcRn was demonstrated in xenograft studies, which resulted in a better efficacy than the wild-type version [49].

## 5. Conclusions

Our first-in-human study results indicate that ARGX-111 is safe in advanced cancer patients, and the drug showed signs of therapeutic activity in MET-overexpressing tumors as well as *MET*-amplified tumors based on its unique Modes of Action, thereby supporting further clinical development of the antibody as monotherapy or in combination.

## Figures and Tables

**Table 1:** Patient demographics and baseline characteristics.

	Q3W					Q2W
	0.3 mg/kg	1 mg/kg	3 mg/kg	10 mg/kg	Total	3 mg/kg
**N**	2	2	12	3	19	5
**Age**						
Median	60	52	60	47	60	57
Range	49–71	41–63	46–70	28–69	28–71	42–79
**Gender**						
Male	1	2	7	1	11	4
Female	1	0	5	2	8	1
**Race**						
Caucasian	2	2	12	3	19	5
**ECOG**						
Grade 0	0	2	4	0	6	1
Grade 1	2	0	8	3	13	4
**Primary cancer type**						
Stomach	1	0	0	0	1	2
Esophagus	0	2	1	0	3	0
Cervix	0	0	1	1	2	0
Pancreas	1	0	1	0	2	0
Lung	0	0	2	0	2	1
Breast	0	0	1	0	1	0
Bile duct	0	0	1	1	2	0
Kidney	0	0	2	1	3	2
Vagina	0	0	1	0	1	0
Bladder	0	0	1	0	1	0
Bone	0	0	1	0	1	0

N, number of patients; ECOG, Eastern Cooperative Oncology Group.

**Table 2:** Summary of treatment-emergent adverse events (TEAEs) in ≥20% of all patients (dose escalation plus safety expansion) by preferred term (PT).

TEAE by PT	Any Graden, N (%)	Grade ≥ 3 n, N (%)
IRR	58, 19 (79)	2, 2 (8)
Fatigue	20, 13 (54)	1, 1 (4)
Constipation	10, 10 (42)	0, 0 (0)
Nausea	11, 8 (33)	0, 0 (0)
Decreased appetite	11, 8 (33)	0, 0 (0)
Myalgia	8, 8 (33)	0, 0 (0)
Vomiting	7, 7 (29)	0, 0 (0)
General physical health deterioration	7, 7 (29)	6, 6 (25)
Arthralgia	5, 5 (21)	1, 1 (4)

n, number of events; N, number of patients; %, percentage of patients; IRR, infusion-related reaction.

**Table 3:** Pharmacokinetics of ARGX-111 at cycle 1 after the first ARGX-111 administration (actual time points).

	Q3W				
		0.3 mg/kg	1 mg/kg	3 mg/kg	10 mg/kg
**Cmax (µg/mg)**	N	2	2	12	3
	AM	5.65	14.2	30.3	76.5
	Sd	1.51	0.07	17.4	64.6
	median	5.65	14.2	25.2	101
**AUC_0-t_ (µg·h/mL)**	N	2	2	12	3
	AM	68.2	481	3020	4500
	Sd	0.81	309	1780	4420
	median	68.2	481	2620	4610
**AUC_0-∞_ (µg·h/mL)**	N	NA	1	11	2
	AM	NA	882	3580	7690
	Sd	NA	NA	1910	3320
	median	NA	882	8050	
**CL (** **mL/h)**	N	NA	1	11	2
	AM	NA	0.07	0.08	0.09
	Sd	NA	NA	0.04	0.02
	median	NA	0.07	0.08	0.09
**V_d_ (L)**	N	NA	1	11	2
	AM	NA	6.79	11.5	14.3
	Sd	NA	NA	4.33	7.22
	median	NA	6.79	10.8	14.3
**t_½_ (h)**	N	NA	1	11	2
	AM	NA	63.8	116	112
	Sd	NA	NA	54.6	72
	median 0	NA	63.8	102	112

N, number of patients; C_max_, maximum concentration; AM, arithmetic mean; sd, standard deviation; AUC_0-t_, area under the plasma concentration–time curve from time zero to the last measured concentration; AUC_0-∞_, area under the plasma concentration–time curve from time zero to infinity; CL, = clearance; V_d_, apparent volume of distribution; t_½_, half-life.

## Data Availability

The data supporting this study will not become publicly available.

## Supplementary Materials

The following are available online at https://www.mdpi.com/article/10.3390/biomedicines9060665/s1, Table S1: MET amplification by FISH and MET expression by IHC in archived FFPE biopsies from patients included in the SE phase, Table S2: NK cell count and function in the various ARGX-111 dose cohorts, Figure S1: MET positivity as measured by IHC (expression) and FISH (MET gene amplification), Figure S2: Immunogenicity of ARGX-111, Figure S3: Representative PET/CT scans of a MET-amplified renal cell cancer patient showing stabilization of disease to ARGX-111. Figure S4: Schematic flow chart for the PET/CT imaging procedure adopted in the dose escalation phase.
