# Supplementary material for: The NHance® Mutation-Equipped Anti-MET Antibody ARGX-111 Displays Increased Tissue Penetration and Anti-Tumor Activity in Advanced Cancer Patients
Source: Biomedicines. 2021 Jun 10;9(6):665. doi: 10.3390/biomedicines9060665 (PMC8229762; doi:10.3390/biomedicines9060665)
Supplement: Supplementary file 1 [file biomedicines-09-00665-s001.zip › biomedicines-1193363-supplementary.pdf]

Supplementary material

Table S1. ***MET*** **amplification by FISH and MET expression by IHC in archived FFPE biopsies from patients included in the SE phase.**

	FISH		IHC		
**Primary tumor site**	***MET/CEP7***	**MET copies**	**% MET+**	**intensity**	**subcel. loc**
kidney	2.45	8.14	100	3+	M>C
lung	2.42	3.82	5	0	C
stomach	3.43	8.44	95	2+	C
kidney	2.70	7.50	80	3+	C
stomach	2.46	6.21	85	3+	M>C

M, membrane; C, cytoplasm

.

Table S2. NK cell count and function in the various ARGX-111 dose cohorts.

Cycle	Assessment		0.3 mg/kg (N = 2)	1 mg/kg (N = 2)	3 mg/kg (N = 12)	10 mg/kg (N = 3)
C1D1	NK count	N	2	2	12	3
		mean	142	85.5	250	72.7
		sd	198	12.0	239	27.6
	Specific lysis (%)	mean	6.55	12.3	27.2	22.3
		sd	5.30	25.0	20.4	10.5
C2D1	NK count	N	1	2	11	2
		mean	3.00	101	255	90.5
		sd	N/A	65.1	158	3.54
	Specific lysis (%)	mean	18.8	9.95	20.1	24.3
		sd	N/A	12.4	13.9	28.4
C3D1	NK count	N	1	1	6	0
		mean	3.00	181	201	N/A
		sd	N/A	N/A	76.3	N/A
	Specific lysis (%)	mean	23.9	7.70	19.9	N/A
		sd	N/A	N/A	6.48	N/A
C4D1	NK count	N	1	1	6	0
		mean	12.0	37.0	188	N/A
		sd	N/A	N/A	80.8	N/A
	Specific lysis (%)	mean	29.0	0.30	10.8	N/A
		sd	N/A	N/A	9.29	N/A

NK count: CD16+/CD56+10E3/ml. Legend: sd, standard deviation; N/A, not applicable

.

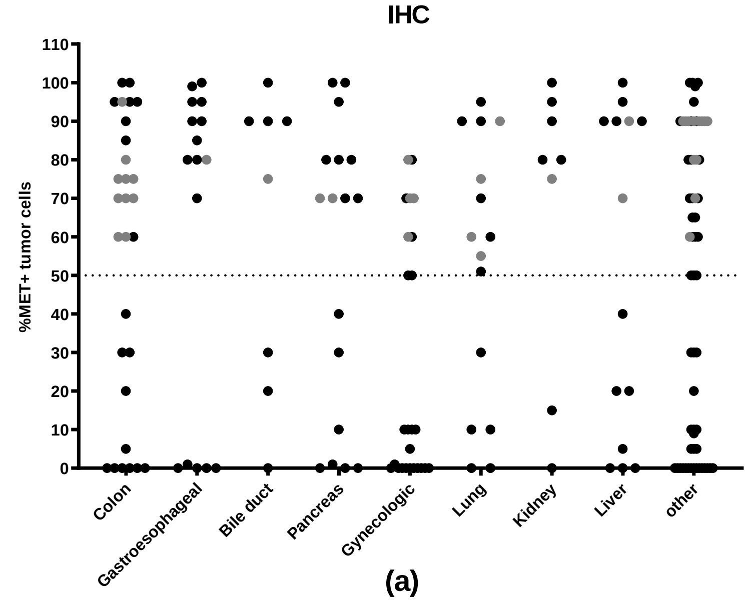

(a)

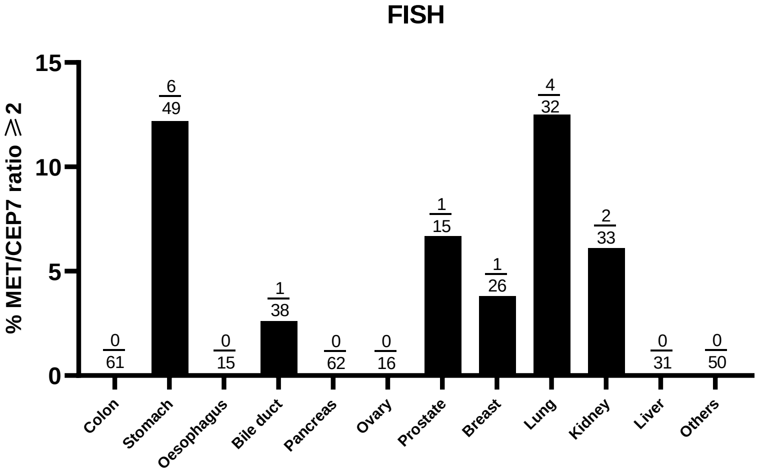

(b)

Figure S1. MET positivity as measured by IHC (expression) and FISH (*MET* gene amplification). (**a**) Percentage of tumor cells expressing MET as determined by IHC in 192 biopsies pre-screened for the DE phase. Grey dots indicate samples that display a percentage of cells expressing MET > 50% and a MET expression intensity < 2+ (not meeting the inclusion criteria). (**b**) Percentage of tumors positive for *MET* gene amplification (*MET*/*CEP7* ratio ≥ 2) as determined by FISH in 428 tumor samples pre-screened for the SE phase. Data are clustered by primary tumor site. The fraction of FISH-positive samples for each tumor type is indicated on the right.

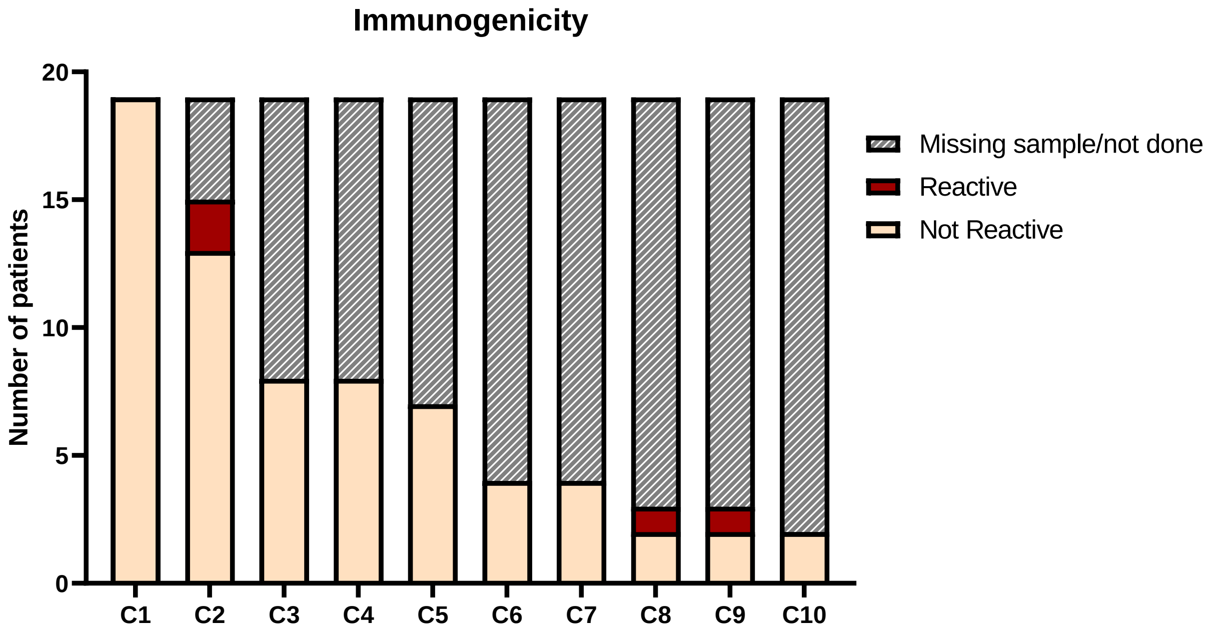

Figure S2. Immunogenicity of ARGX-111. Number of patients in the DE with ADA reactive samples, not reactive samples or missing data (not done) up to Cycle 10.

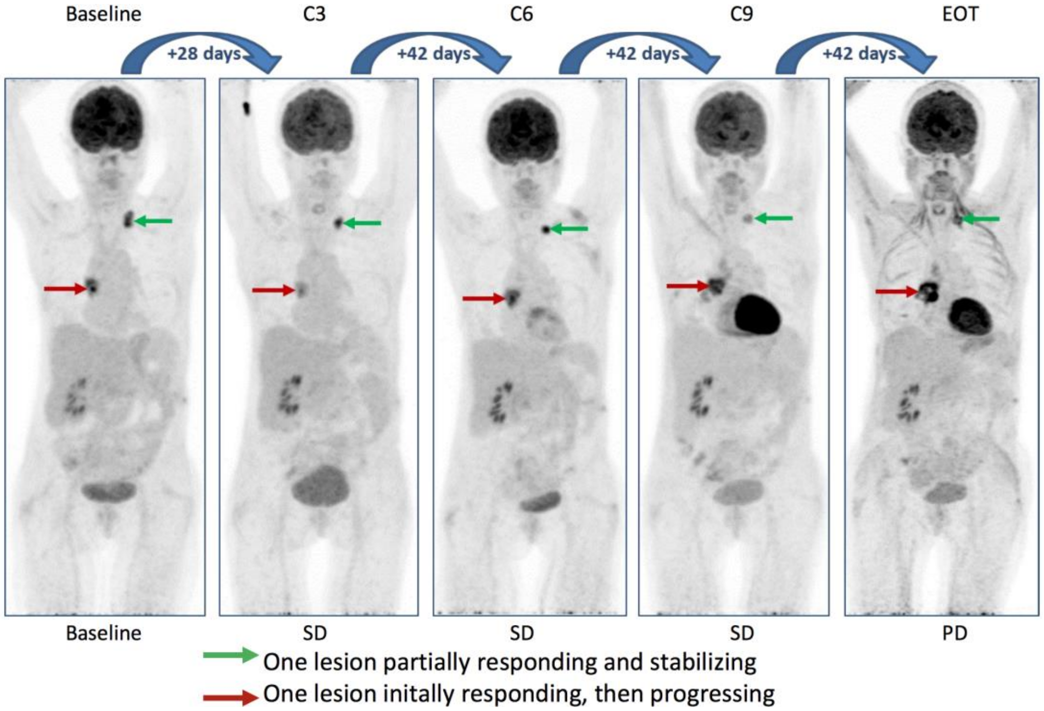

Figure S3. Representative PET/CT scans of a *MET* amplified renal cell cancer patient showing stabilization of disease to ARGX-111.

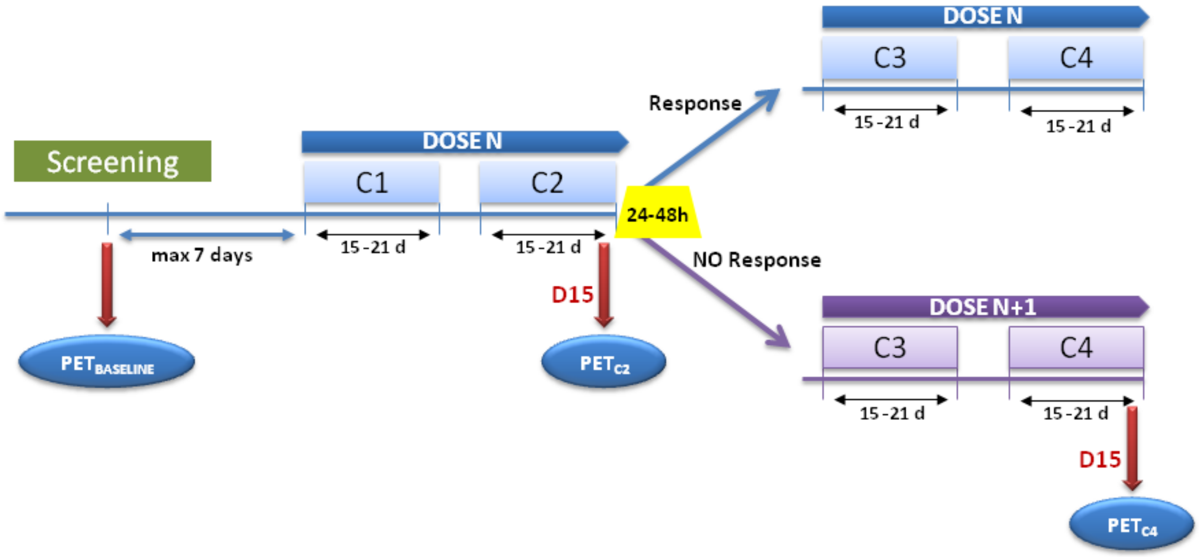

Figure S4. Schematic flow chart for the PET/CT imaging procedure adopted in the dose escalation phase. All patients underwent a baseline PET/CT scan during screening in the week before first drug administration. A second scan was performed at C2D15 during the treatment period and compared to baseline scan. Patient demonstrating stable or increased metabolic activity (no response) were escalated to the next dose level from C3D1. A third scan was performed on escalated patients on C4D15. Results were reviewed by two independent nuclear medicine physicians.
